# Estimates of Particulate Organic Carbon Flowing from the Pelagic Environment to the Benthos through Sponge Assemblages

**DOI:** 10.1371/journal.pone.0029569

**Published:** 2012-01-04

**Authors:** Alejandra Perea-Blázquez, Simon K. Davy, James J. Bell

**Affiliations:** School of Biological Sciences, Victoria University of Wellington, Wellington, New Zealand; National Institute of Water & Atmospheric Research, New Zealand

## Abstract

Despite the importance of trophic interactions between organisms, and the relationship between primary production and benthic diversity, there have been few studies that have quantified the carbon flow from pelagic to benthic environments as a result of the assemblage level activity of suspension-feeding organisms. In this study, we examine the feeding activity of seven common sponge species from the Taputeranga marine reserve on the south coast of Wellington in New Zealand. We analysed the diet composition, feeding efficiency, pumping rates, and the number of food particles (specifically picoplanktonic prokaryotic cells) retained by sponges. We used this information, combined with abundance estimates of the sponges and estimations of the total amount of food available to sponges in a known volume of water (89,821 m^3^), to estimate: (1) particulate organic carbon (POC) fluxes through sponges as a result of their suspension-feeding activities on picoplankton; and (2) the proportion of the available POC from picoplankton that sponges consume. The most POC acquired by the sponges was from non-photosynthetic bacterial cells (ranging from 0.09 to 4.69 g C d^−1^ with varying sponge percentage cover from 0.5 to 5%), followed by *Prochlorococcus* (0.07 to 3.47 g C d^−1^) and then *Synechococcus* (0.05 to 2.34 g C d^−1^) cells. Depending on sponge abundance, the amount of POC that sponges consumed as a proportion of the total POC available was 0.2–12.1% for Bac, 0.4–21.3% for Prochlo, and 0.3–15.8% for Synecho. The flux of POC for the whole sponge assemblage, based on the consumption of prokaryotic picoplankton, ranged from 0.07–3.50 g C m^2^ d^−1^. This study is the first to estimate the contribution of a sponge assemblage (rather than focusing on individual sponge species) to POC flow from three groups of picoplankton in a temperate rocky reef through the feeding activity of sponges and demonstrates the importance of sponges to energy flow in rocky reef environments.

## Introduction

The trophic relationships between benthic and pelagic communities mainly depend on the movement of primary production in surface waters to deeper layers [1]. In highly productive marine areas, the major biological factors structuring benthic communities are recruitment and the flow of organic matter from the pelagic domain to the benthos [2]. High water motion in coastal zones increases the flow of nutrients between pelagic and benthic environments making the study of benthic trophodynamics (i.e. the flow of energy and particles) important for understanding the dynamics of coastal systems [3]. Furthermore, benthic marine food webs are essential biological components of coastal ecosystems because of their role in organic matter cycling and because they provide a link between the water column, benthic organisms and sediments [4].

Suspension-feeding is one of the most widespread feeding strategies among benthic organisms including members of the Porifera, Cnidaria, Bryozoa, Brachiopoda, Annelida (Polychaeta), Mollusca (Bivalvia), Echinodermata, Crustacea and Tunicata [5]. Suspension-feeding invertebrates play an important role in the flow of carbon through marine ecosystems as they have the ability to control the cycling of nutrients, organic matter, plankton and detritus [6], [7], [8], and move carbon from the pelagic environment to the benthos (and *vice versa*). Benthic-suspension feeders are considered among the most efficient organisms at extracting and processing energy from marine ecosystems [7] and the trophic strategies of these organisms are strongly related to the availability of carbon occurring in the water column [2]. Hence, studying the feeding ecology of these organisms is important for understanding the dynamics of particles in the water column and energy flow in marine ecosystems. Sponges are one of the most important components of the suspension-feeding community in rocky environments, as they are very abundant and are able to effectively exploit pelagic food resources. Sponges therefore provide coupling between primary production and the benthos by converting planktonic carbon into sponge biomass [9], [10]. This carbon can then be used by higher trophic levels through the consumption of sponge biomass by organisms such as fish, sea stars [11], turtles [12], [13], sea urchins [14], [15] and opisthobranchs [16]. Alternatively, sponges may act as a carbon sink, since many species are unpalatable to potential predators and long-lived [17], [18].

Both photoautotrophic and heterotrophic picoplankton are important components of global marine primary production [19], [20] since they are major participants in global carbon cycles [20]. The eukaryotic forms of the picoplankton can also play an important role in the generation of primary production in marine coastal waters [21], [22]. Photoautotrophic picoplankton (<2 µm in size) are single-celled free-living cyanobacteria in the water column dominated by two genera; *Prochlorococcus* and *Synechococcus* [23], [24]. These organisms occupy key positions at the base of marine food webs, and their abundance and productivity potentially dictate the flow of carbon through food webs [25]. The carbon sequestered as a result of photosynthesis is moved to higher trophic levels via intermediate small grazers, such as flagellates [26] and ciliates [27], which are most likely major consumers of *Prochlorococcus* and *Synechococcus*; this represents an additional trophic link between picoplankton primary producers and higher trophic levels [27]. *Prochlorococcus* and *Synechococcus* cells are too small to be consumed directly by other components of the plankton such as small copepods and cladocerans. However, they are a significant food resource for larger benthic suspension-feeding organisms such as bivalves, ascidians and sponges [8], [28].

Previous research has demonstrated that sponges efficiently feed on picoplankton including *Prochlorococcus*, *Synechococcus* and bacterial cells, and are capable of moving large quantities of these organisms from the pelagic environment to the benthos [29], [30]. In addition, species-level studies of plankton removal by sponges and their role in bottom-up effects [10], [31] have shown that sponges are significant sinks for particulate organic material (POM) and for dissolved organic carbon (DOC) [32]; and recently, a study has provided direct evidence for the utilisation of dissolved organic matter (DOM) by sponges [33]. Previous studies have examined the natural diet of temperate demosponges using different *in situ* techniques. However, these have only been conducted on a small number of species [28], [29], [31], [34] and the ecosystem-level effects of sponge feeding have not yet been estimated.

Recent reviews on the functional roles that sponges play in marine systems [35], [36] have highlighted the ecological importance of sponges, particularly in habitats where they occur in high densities. Despite their potentially important interaction with the water column, many aspects of sponge biology and ecology remain poorly described, and as a result our overall understanding of the energy transfer from pelagic to benthic habitats resulting from feeding by sponge assemblages, remains poorly understood. Our study determined the ecological importance of a temperate sponge assemblage with respect to its use of the particulate organic carbon (POC) fraction in the water column, by measuring rates of carbon consumption in the form of particulate matter (specifically picoplanktonic particles and from hereon termed POC). We combined this information with data on the following characteristics of the sponge species: diet composition, sponge abundance, feeding efficiency, pumping rate and particles removed (number of cells removed per ml min^−1^), to estimate the proportion of the available standing stock of POC in the water column of a defined coastal region that was being consumed by the sponge assemblage.

## Results

### Cell concentrations and retention efficiency

**Figure 1:**
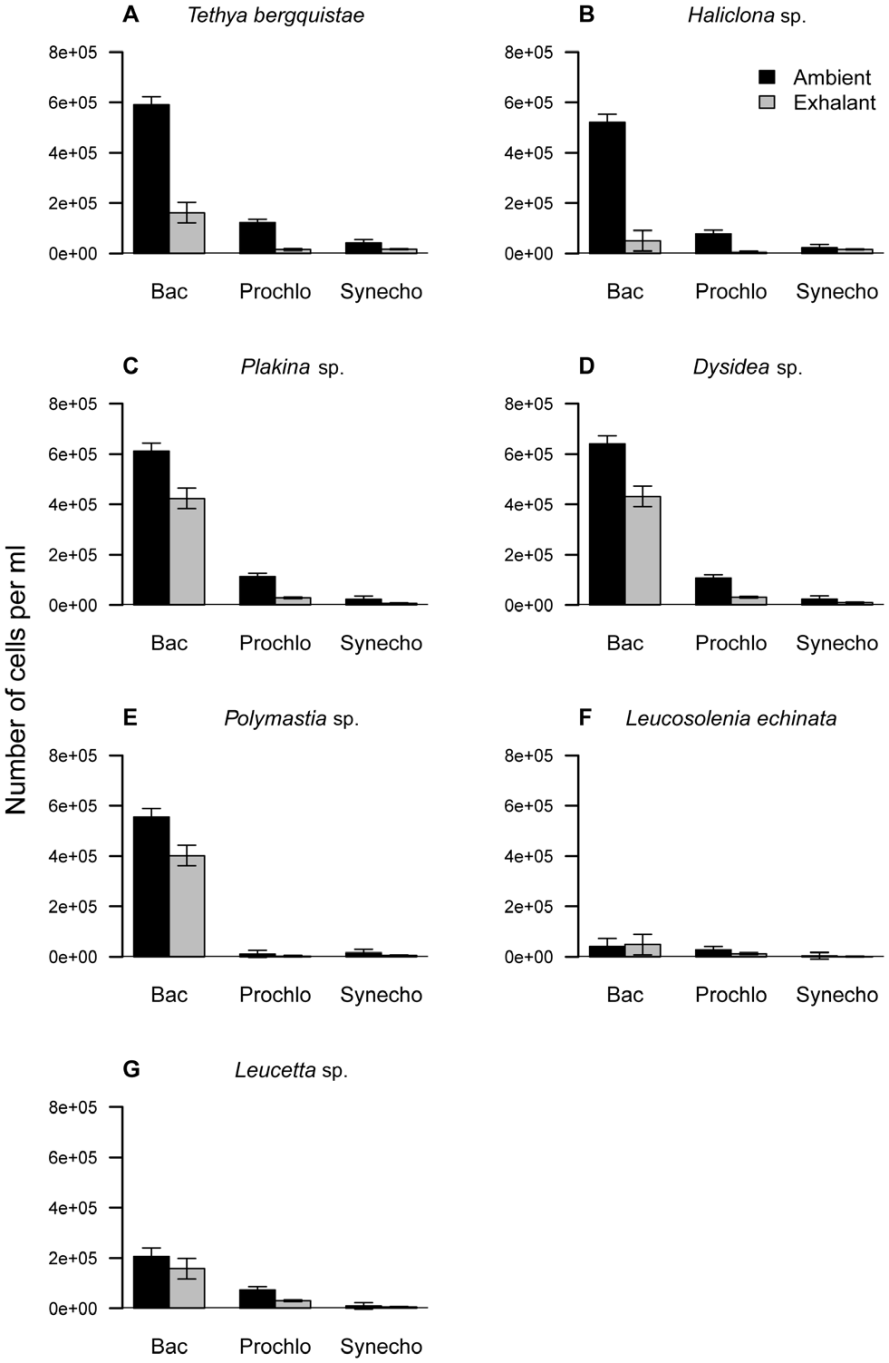
Inhalant versus exhalant cell concentrations and types of picoplankton for each of the study species. Detailed legend: Inhalant versus exhalant cell concentrations (no. of cells ml^−1^) and types of picoplankton (non-photosynthetic bacteria –Bac, *Prochlorococcus* –Prochlo, *Synechococcus* –Synecho) for **A**, *Tethya bergquistae*; **B**, *Haliclona* sp.; **C**, *Plakina* sp.; **D**, *Dysidea* sp.; **E**, *Polymastia* sp.; **F**, *Leucosolenia echinata*; **G**, *Leucetta* sp.

Three populations of picoplanktonic organisms were identified: Bac, Prochlo and Synecho-type cyanobacteria that sponges removed from the ambient (inhalant) water. Bac were the most abundant picoplanktonic cells, followed by Prochlo- and then Synecho-types. The average ambient cell concentration of Bac was markedly higher (4.4±2.7×10^5^ cells ml^−1^) than that of Prochlo (7.2±4.6×10^4^ cells ml^−1^) and Synecho (1.9±1.5×10^4^ cells ml^−1^). The range of cell concentrations measured in the water surrounding the different species are presented in Table 1. The GLM analysis of inhalant versus exhalant cell concentration and types of picoplankton yielded significant differences between the concentrations of cells found in the inhalant and exhalant currents for all of the study species (Fig. 1), demonstrating the retention or removal of food particles by the sponge species. In general, the concentration of picoplanktonic organisms found in the ambient current of all the sponges remained similar throughout the sampling period (Fig. 1). The large number of picoplanktonic cells that sponges can filter on a daily basis in a known volume of water are summarised in Table 2. All sponge species removed the three types of picoplankton found in the ambient water with an overall removal efficiency of 40±14% for Bac, 72±11% for Prochlo, and 54±18% for Synecho. The ranges of removal efficiency for the different picoplanktonic particles and sponge species are presented in Table 2.

**Table 1:** Ranges of ambient cell concentrations.

Species	Number of cells in ambient water per ml^−1^		
	Bac	Prochlo	Synecho
*Dysidea*	7×10^5^–8.3×10^5^	1×10^5^–1.2×10^5^	2.1×10^4^–2.8×10^4^
*Haliclona*	4.6×10^5^–5.7×10^5^	7.5×10^4^–8.4×10^4^	2.1×10^4^–2.3×10^4^
*Leucetta*	6×10^4^–1.6×10^5^	4.7×10^4^–5.3×10^4^	5.9×10^3^–7×10^3^
*Leucosolenia*	5×10^4^–6.6×10^4^	1.8×10^4^–2×10^4^	2.5×10^3^–2.7×10^3^
*Plakina*	3.4×10^5^–1×10^6^	8.6×10^4^–1.6×10^5^	1.7×10^4^–3.4×10^4^
*Polymastia*	5×10^5^–6.1×10^5^	5.8×10^3^–1.4×10^4^	6.5×10^3^–2.2×10^4^
*Tethya*	5.3×10^5^–6.3×10^5^	9.9×10^4^–1.5×10^5^	2.4×10^4^–6.8×10^4^

The cell numbers are for the three types of picoplankton measured in the water surrounding the different study species.

**Table 2:** Estimated mean flow rate, amount of water filtered and picoplanktonic cells removed by the study species over the sampling period.

Species	Flow rate	Number of cells removed/ml/min		
	(ml min^−1^)	Bac	Prochlo	Synecho
*Dysidea*	75.8±50.5	1.77×10^7^±1.97×10^7^	6.47×10^6^±6.02×10^6^	1.09×10^6^±9.45×10^5^
*Haliclona*	40.5±20.6	1.88×10^7^±9.02×10^6^	3.00×10^6^±1.55×10^6^	2.67×10^5^±1.62×10^5^
*Leucetta*	76.0±30.6	3.40×10^5^±1.57×10^5^	2.22×10^6^±9.27×10^5^	2.20×10^5^±4.28×10^4^
*Leucosolenia*	32.6±8.0	7.31×10^5^±9.37×10^5^	3.37×10^5^±1.81×10^5^	6.00×10^4^±1.20×10^4^
*Plakina*	45.5±22.9	1.16×10^7^±1.67×10^7^	3.49×10^6^±1.54×10^6^	7.16×10^5^±4.07×10^5^
*Polymastia*	110.4±36.5	1.84×10^7^±1.89×10^7^	1.04×10^6^±7.36×10^5^	1.31×10^6^±1.13×10^6^
*Tethya*	111.4±11.7	4.78×10^7^±6.57×10^6^	1.18×10^7^±1.80×10^6^	2.81×10^6^±2.85×10^6^

Flow rate is the volume of water filtered by the sponge considering the total number of oscula from three specimens of each species. Data presented are averages (± StdDev), calculated for three specimens of each sponge species.

### Volume flow rate pumped by the study species

*T. bergquistae* had the highest flow rate of all the study species (111.4±11.7 ml min^−1^), followed by *Polymastia* sp., with an average flow rate of 110.4±36.5 ml min^−1^; the lowest flow rate measured was for *L. echinata* (32.6±8.0 ml min^−1^) (Table 3). The retention efficiency, volume flow rate, and ambient concentration of each picoplanktonic particle were used to calculate the number of cells removed (ml min^−1^) by each species (Table 3). All these values were used to estimate the amount of carbon acquired by the different sponge species from the picoplanktonic organisms they retained.

**Table 3:** Ranges of retention efficiency for the three types of picoplankton removed by the study species.

Species	Retention efficiency		
	Bac	Prochlo	Synecho
*Dysidea*	9–42%	54–96%	36–73%
*Haliclona*	88–91%	93–95%	18–43%
*Leucetta*	3–5%	56–59%	41–49%
*Leucosolenia*	0–66%	37–67%	66–75%
*Plakina*	9–41%	59–89%	51–81%
*Polymastia*	13–50%	52–91%	7–89%
*Tethya*	67–85%	83–94%	31–82%

### Picoplankton biomass retained and carbon acquired by sponges

**Figure 2:**
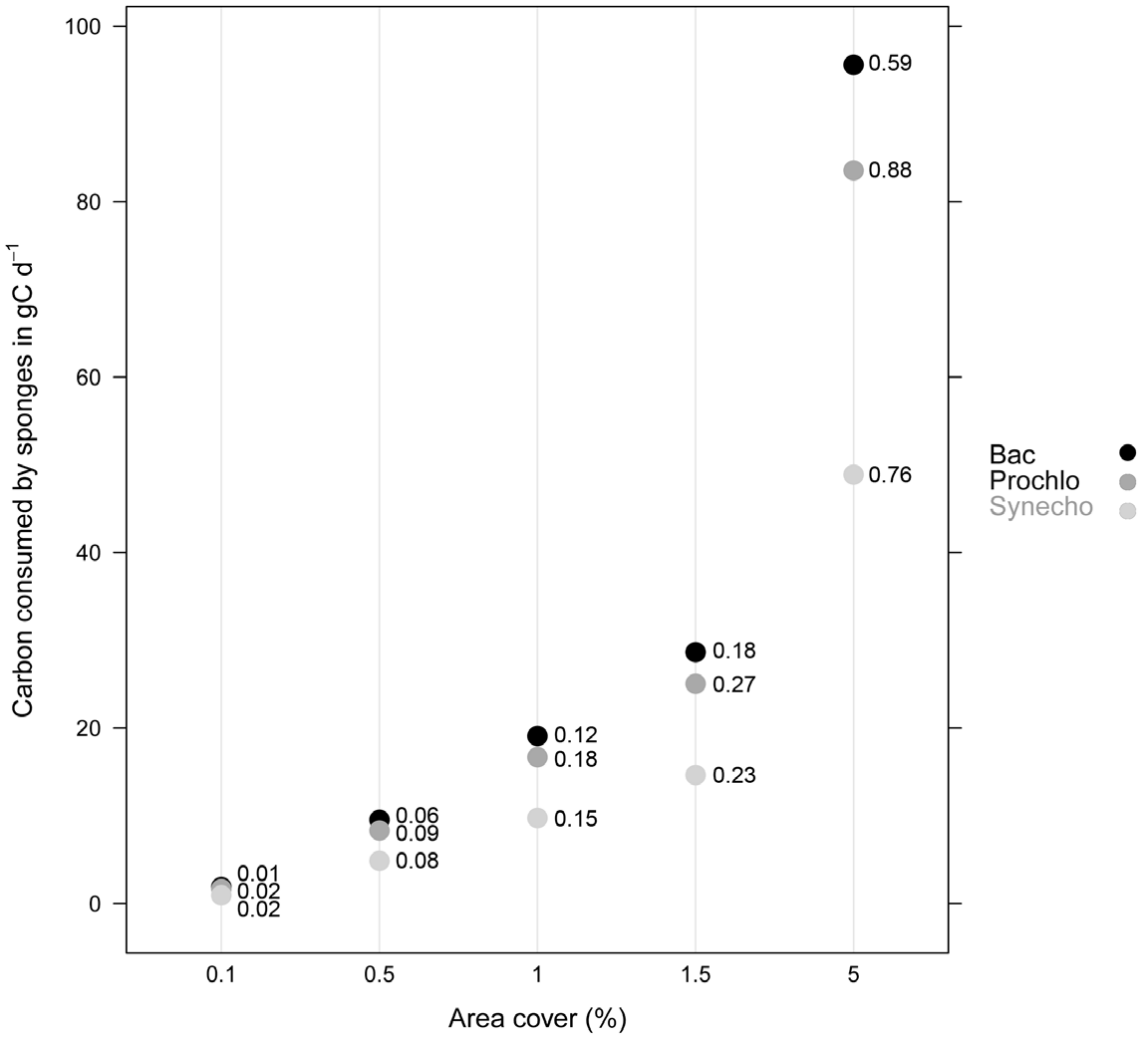
Carbon consumed by sponges from the picoplanktonic organisms retained in the study area. Detailed legend: Carbon consumed by sponges (gC d^−1^) in the area of the Marine Reserve from the three types of picoplanktonic organisms they retain. The graph shows a range of values for sponge percentage cover (0.1, 0.5, 1, 1.5 and 5%) measured at the site, as well as the percentage of POC consumed from the available POC within the MR. The values of the percentage of POC consumed by sponges from the total available in the MR are the numbers next to the black and grey dots.

Using habitat maps, the subtidal rocky reef area (considered the habitable substrate for sponges) within the study area was estimated to be 3.02 km^2^, and the estimated volume of water in the area (calculated from bathymetry data) was 89,821 m^3^ at high tide. Based on these figures and using the data from Equation 1, the total number of picoplanktonic cells present in the water column of the study area at any point in time was calculated as: Bac = 4.0±2.5×10^16^ cells, Prochlo = 6.4±4.1×10^15^ cells, and Synecho = 1.7±1.3×10^15^ cells. In a similar way, using Equation 2 the number of cells that the sponge assemblage would be capable of removing on a daily basis in the study area was estimated and the results are presented in Table 4. Using equation 3 we were able to calculate the average amount of POC consumed for the different values of sponge coverage in the study area per day. The results from these calculations showed that in terms of POC, Bac were the primary carbon source for all sponges followed by Prochlo and then Synecho (Table 4). Finally, the percentage of POC consumed by sponges from the total available prokaryotic POC in the form of each picoplanktonic organism in the study area was estimated using Equation 4. The results are presented in Figure 2 for the different estimates of sponge abundance. The graph shows a range of values for sponge percentage cover (0.1, 0.5, 1, 1.5 and 5%) measured at the site, as well as the percentage of POC consumed from the total available POC (considering the three types of picoplankton) within the MR (Fig. 2). Assuming a low sponge cover (1%), an assemblage would consume 0.2% of the total available POC in the form of Bac, 0.4% of Prochlo and 0.3% of Synecho per day. However, when assuming a high sponge cover (5%), an assemblage would consume 12.1% of the total POC available in the form of Bac, 21.3% of Prochlo and 15.8% of Synecho per day in the study area.

**Table 4:** Summary of the number of cells filtered by sponge assemblages from each type of picoplankton retained.

Sponge cover (%)	Picoplankton (cells)		
	Bac	Prochlo	Synecho
0.1	2.13×10^18^	5.25×10^17^	1.19×10^17^
0.5	1.07×10^19^	2.62×10^18^	5.97×10^17^
1.0	2.13×10^19^	5.25×10^18^	1.19×10^18^
1.5	3.20×10^19^	7.87×10^18^	1.79×10^18^
5.0	1.07×10^20^	2.62×10^19^	5.97×10^18^

Values (number of cells filtered, cells d^−1^) were calculated using a range of estimated abundances of sponge percentage cover in the study area.

## Discussion

In recent years, there has been an increasing interest in the role that benthic suspension-feeders play in the flow of energy between the water column and the benthos. However, most of these studies have focused on coral reefs [37], [38] and polar ecosystems [39], [40], [41], with less attention being given to temperate regions [42]. Interestingly, fluxes in temperate and polar systems have been estimated to be higher than in tropical systems most likely because of higher productivity of the pelagic ecosystems in these regions [9], [18]. Since sponges are usually a dominant group (with the exception of corals on reefs) across hard substratum habitats worldwide, there have been a growing number of studies that have quantified the carbon flow as a result of sponge feeding activities. Previous studies based on *in situ* measurements of individual sponge species in temperate regions, have estimated carbon fluxes of 29 mgC m^2^ d^−1^ in the sponge *Mycale lingua* [43], and 3.5 mgC m^2^ d^−1^ in the sponge *Callyspongia* sp. [31] from prokaryotic organisms. However, our study is the first to estimate the contribution of a sponge assemblage (rather than focusing on individual sponge species) to the POC flow in a temperate rocky reef through the sponges' *in situ* feeding activities. Although there are more than just the seven species that we examined in the study area (50 in total have been reported), the study species selected are by far the most abundant and represent >80% of the available biomass. Therefore, we believe our data provides a reasonable representation of POC consumption by the sponge assemblage. Furthermore, we would expect that these other species are likely to feed within the range of retention efficiencies as the sponges we studied, therefore their occurrence is taken into account through our approach of using a range of overall sponge abundance values. We found that the POC consumption by sponges based on prokaryotes ranged from 70 to 3,500 mgC d^−1^ based on the minimum and maximum area estimates of 1% and 5% m^2^ of sponge cover, respectively. The percentage of the total amount of POC in the water column consumed by sponges ranged from 0.01% to 0.70% based on the same area estimates of sponge cover, respectively. To place these values in context, a study carried out in a shallow coastal region of the northern Wadden Sea by Baird et al. [44], estimated that the total phytoplankton consumption by suspension-feeders was 387 mgC m^2^ d^−1^ (based on a total area of 270 km^2^). In the present study, a much smaller area (3.02 km^2^) was considered and did not take into account other suspension-feeders (e.g. cryptic sponges, ascidians or bryozoans) that are present on the rocky reefs. Despite this, the values estimated in our study are higher. Therefore, we suggest that sponge assemblages have the potential to play a very important role in the transfer of POC from the water column to the benthos in the temperate marine ecosystem studied here, and potentially in other temperate sites.

The ability of sponges to efficiently capture other types of plankton (<10 µm) such as pico- and nanoeukaryotes has been documented [30], [34], [45], and some authors have been able to distinguish different populations of viruses in natural seawater samples and their removal by sponges [46], [47]. While we only considered picoplankton in this study, other important possible sources of carbon for sponges include DOM and DOC [33], [48], as well as other forms of POC, from both live (i.e. planktonic organisms) and detrital sources [49]. Yahel *et al.* [32] found considerable DOC uptake (more than 90% of their daily intake) by a sponge from the Red Sea, and De Goeij *et al.* [48] found similar high levels of DOC uptake by several more sponge species in the Caribbean. Symbiotic microorganisms constitute another potential food source for sponges, either by direct consumption [50], or by phototrophy where the cyanobacterial symbionts in some sponges supply a high percentage of their carbon budget [51], [52]. It is likely that the sponges examined in our study are exploiting a number of different food sources to meet their overall carbon requirements. The values presented in this study on the contribution of the use of picoplankton as a carbon source, appear to constitute only a fraction of the total energy (carbon) budget of the study species [53], and these other sources will be the focus of future study to provide a complete carbon budget for this region.

### Carbon flow through sponge assemblages in a defined study area

The results from this study indicate that sponge assemblages in the rocky reef studied feed more efficiently on smaller cells (Bac) than on bigger cells (Prochlo and Synecho). Although Bac were not retained as efficiently as the larger types of picoplankton (e.g. Prochlo), their higher concentration in the water column meant that they contributed more to total C uptake, without reflecting efficiency *per se*. Bac contributed the most to sponge diets with a C uptake of 95.6 g C d^−1^, compared to the carbon uptake from Prochlo (83.6 g C d^−1^) and Synecho (48.9 g C d^−1^). Thus, it seems that for the sponge species studied here the relative importance of different picoplanktonic organisms as a food source is determined foremost by the concentration of these organisms in the surrounding water within which sponges are living [54], and secondarily by their ability to retain them. The findings from this study are in accordance with previous studies where Bac have been found to be one of the primary sources of energy (in the form of particulate carbon) for sponges [10], [55], [56]. In the present study a range of sponge abundance figures were used, since we know there are high levels of variability in sponge abundance across the entire study area, therefore it is difficult to accurately estimate overall sponge abundance across the entire study site. However, it is worth noting that in some areas of the reserve, sponge cover can reach >50%, so POC consumption could be much higher at local scales, potentially even causing localised depletion.

The cell concentrations of the different picoplanktonic populations in the ambient water are similar to other studies that have investigated sponge feeding in temperate regions [34], [43], [56], [57]. Previous studies have confirmed temporal variation in pumping rates [58], [59], though this appears to be species dependent [60]. This is supported by the results presented here where different pumping rates were measured for different sponge species. It is noteworthy mentioning that our assumption of continuous pumping activity over a 24 h period, is supported by recent findings by Pfannkuchen *et al* [61] who detected permanent pumping activity for several sponges *in situ*, using the method of tracer application for the detection of active pumping in sponges, which does not disturb the sponges and is free from experimental artefacts.

### Sponges as trophic links in food webs

Carbon flows through food webs and can also be exchanged with the atmosphere [62]. The driving force of the carbon cycle is the primary production of organic matter by phytoplankton, which is essentially controlled by light intensity and the availability of nutrients [63]. In marine food webs, bacteria are responsible for the recycling of nutrients to primary producers through the so-called ‘microbial loop’ [64], and bacteria in the water column (picoplankton) can be utilised by various groups of suspension-feeders, including sponges. The results from our study confirmed the assumption that sponge feeding represents a significant biomass link between benthic and pelagic habitats. Furthermore, the results suggest that the fluxes of POC provided from the microorganisms they filter place sponges within an important functional group of organisms that link the pelagic microbial food web to the benthos [65], [66], [67].

In this study, the combined characteristics of the seven sponge species analysed were extrapolated to a defined study area, and the estimated volume of water (89,821 m^3^), as well as the rocky reef area (3.02 km^2^), allowed us to calculate the proportion of the available picoplanktonic POC that sponge assemblages have the potential to consume in this area. Because sponges are found worldwide and in high abundances in most hard substratum habitats, these organisms must be included in all energy flow models or food-web networks. This is important, since in some of these models, suspension-feeders provide an essential pathway for energy flow [41], [68]. The construction of such food-web models gives quantitative information on the species and communities involved in marine systems, as well as their rates of consumption and production, dietary composition, and the flow of energy and materials between the system components [41]. These models can then be incorporated in conservation, restoration, and management programmes. The data obtained from this study including prokaryotic biomass consumption (mg C m^−2^), diet composition, POC flow, and the feeding ecology of sponges, could be incorporated in these models in the future. This information will be important for future studies examining the ecological functioning of marine ecosystems, since understanding how changes in primary production or temperature impact ecosystems requires reliable models based on realistic representations of energy fluxes through ecosystems [69].

Since sponges play an important role in the balance and dynamics of carbon and nutrients in the water column [70], the results from this study represent an important step in developing a better understanding of the ecology of sponge-dominated assemblages on subtidal rocky reefs. Furthermore, this study shows that sponge assemblages are important components in temperate rocky habitats and that they play a key role in the transfer of POC from the water column to the benthos. This is particularly relevant since sponge feeding within the microbial loop could represent a significant biomass link with sponges being a sink for picoplankton (Bac, Prochlo and Synecho), and the linkages between sponges and the water column may have important implications for determining overall community structure [71].

### Limitations to the estimates of Carbon flow from pelagic to benthic environments

While this study provides the first direct estimates of the contribution of carbon flow from pelagic environments to the benthos through sponges feeding on three dominant types of picoplankton, there are some important assumptions and potential sources of error that should be considered. Firstly, there is the potential for exhalant sponge water samples to be contaminated by ambient water; however, we minimised this effect by using fluorescein dye to check that the area in front of the osculum was the exhalant stream, and it was this stream we were sampling. If the ambient water had been contaminating the samples then we would have expected little difference between the ambient and exhalant water, which is not what we found. Furthermore, any contamination would result in some under-estimation of the amount of picoplankton consumed by sponges. Our estimates do not consider any potential for localized depletion of food resources by sponges and how this might effect the overall carbon consumption by sponges. However, the Wellington South Coast is a highly dynamic environment, and coupled with the fact that we still found high levels of picoplanktonic organisms in the ambient water samples, which were taken close to the sponges (<3 cm away) suggests that localised depletion is unlikely to be a source of error in our calculations; however, in more sheltered environments this might reduce overall carbon consumption by sponges if water is not replenished. It is also important to note that we have only considered three groups of the most important picoplanktonic organisms in our estimates of carbon flow, and sponges are highly likely to be removing carbon in other forms from pelagic environments to the benthos (including DOC and DOM; see above) and therefore the total amount of carbon consumed by sponges will be higher than our estimates and will be a focus of future studies.

We made the assumption that the picoplanktonic organisms in the water column are homogenously distributed in the water column. Our analyses of ambient water samples showed some variation between sampling events, but generally showed a relatively homogenous spatial distribution of picoplanktonic organisms (Fig. 1). In our estimations of total available POC (from picoplankton), we also assumed a homogenous vertical distribution of organisms. Observations of photosynthetic production (authors unpublished data) from the study area suggest little variation in chlorophyll concentrations down to 20 m, and taken in combination with other studies that have shown little variation in chlorophyll concentration to 50 m depth [72] in well-mixed coastal environments, we believe our assumption to be valid. Finally, we only sampled three specimens of each sponge and this sampling was undertaken over a five-month sampling interval; this may account for some of the variation in the data. Increasing the sample size and trying to confine sampling to shorter time period could potentially reduce this variation, however, the extreme environmental conditions prevented this possibility at our study site. Our sampling period covered the spring and summer in New Zealand, which are both likely to be periods of high production, compared to winter. By randomly sampling individuals/species across the time period, we minimised seasonal effects as much as possible.

In conclusion, this is the first study to estimate the contribution of a sponge assemblage (rather than focusing on individual sponge species) to the particulate carbon flow in a temperate rocky reef through sponge feeding activity on three dominant groups of picoplankton. In this study we demonstrated the clear importance of sponges in linking pelagic and benthic habitats, and we suggest that the effective use and substantial consumption of the picoplankton by sponges might help to explain their ecological success and their capacity to reach high biomass in many marine systems.

## Materials and Methods

### Study site and in situ sampling

This study was conducted on the south coast of Wellington in New Zealand within the Taputeranga Marine Reserve (The Sirens, 41°20′58.5″S, 174°45′50.8″E and Mermaids Kitchen, 41°21′60″S, 174°45′47.5″E). This area supports a high diversity and abundance of sponges, and is characterised by having a high abundance of encrusting and massive sponges commonly found on the sides of channels, crevices, boulders, rock walls and overhangs (Berman *et al.* 2008). Seven of the most common and widespread sponge species from the area were selected for this study: *Dysidea* sp., *Haliclona* sp., *Plakina* sp., *Polymastia* sp., *Tethya bergquistae* (Hooper, 1994), *Leucetta* sp., and *Leucosolenia echinata* (Kirk, 1893). These species were chosen because they are very common in the study area and have well defined exhalant oscula that reduce the risk of sampling error, thereby facilitating easier *in situ* water sampling.

### Collection of water samples

Seawater samples were collected *in situ* using SCUBA. Sampling was conducted at high tide. Samples were collected between November 2008 and March 2009. This sampling interval reflects the difficulty of sampling within this study area due to the very dynamic environment. The samples were randomly collected over this period to avoid any potential bias as a result of the length of the sampling interval. There was no biological reason for the length of the sampling period, it reflected the highly dynamic nature of the study site, and the time required to collect the samples, which meant samples were collected over a prolonged period. Three sponge specimens of each species were used for this study. Fluorescein dye was released at the base of each specimen to visually confirm that sponges were actively pumping and to ensure the exhalant water being sampled was not being contaminated by the ambient water. One pair of inhalant and exhalant water samples were taken from each of the specimens that were haphazardly selected on each dive by using 5-ml sterile plastic syringes with blunt-ended needles. The inhalant water of each specimen was sampled by slowly drawing water at a distance of ∼3 cm from the sponge ostia, and the exhalant water was sampled from inside the oscular aperture taking care not to touch the sponge. There are some drawbacks of the use of the syringe method as discussed by Yahel et al. [28]; however, this method has been successfully applied in other studies looking at the diet composition of temperate sponges [43], [73], [74]. To overcome the problems identified by Yahel et al. [28] care was taken to draw the water slowly over the period of several minutes to ensure the exhalant water leaving the sponge was sampled, rather than being sucked from the sponge, and the use of fluorescein dye observations confirmed we were sampling exhalant water. Each sponge specimen was photographed *in situ* next to a ruler to measure the height, width and length to relate area covered to sponge biomass. The number and diameter of all oscula per sponge specimen were recorded and measured with the ruler. This information was combined with oscular flow rate (see below) to provide an estimate of the total amount of water being pumped by the sponge. After collection, water samples were transferred into sterile 1.5 ml cryovials with freshly prepared glutaraldehyde (0.1% final concentration), taken to the laboratory (which is 100 m from the sampling site), frozen in liquid nitrogen and stored at −80°C following the protocol described by Marie *et al.* [75] for natural seawater samples, until the flow cytometric analysis could be performed.

### Flow cytometry and data analyses

In preparation for flow cytometric analysis, samples were thawed to room temperature, then stained in the dark with the DNA-specific dye Hoechst 33342 (0.2 µg ml^−1^ final concentration) for bacterial identification. It is noteworthy that the sponge species studied here were found to feed mainly on picoplankton, and only in a few ambient samples were we able to detect the fluorescence emission of a small percentage (∼0.4–7%) of larger cells (∼5 µm in size), that could possibly be pico and nano-eukaryotic algae). Because of their low percentage and presence only in a few samples, these cells were not included in the subsequent analysis.

Seawater samples were analysed for quantification of non-photosynthetic bacteria and cyanobacterial cells (*Prochlorococcus* spp. “Prochlo” and *Synechococcus* spp. “Synecho”) using a BD LSR II SORP (Special Order Research Product) cytometer equipped with five lasers. The non-photosynthetic microbes detected with the Hoechst staining as DNA containing particles, were considered as bacterioplankton. The use of the term heterotrophic bacteria is common in the literature to describe these DNA containing particles; however, we assigned the operative term “Bac” for these bacterioplankton since we do not know if they are heterotrophic, chemosynthetic or chemoheterotrophic bacteria. Forward scattered light (FSC) was collected using a photodiode and side scattered light (SSC) was collected using a photomultiplier tube (PMT) with a 488 nm band-pass filter (488/10); due to the small size of the micro-organisms, the cytometer was set to trigger off SSC. Identification of all organisms of interest was initially based on the DNA gate (see Perea-Blázquez *et al.* [74] for a detailed description of the flow cytometric method). Synecho cells were identified based on both orange and red fluorescence emission; the phycobiliproteins contained in these organisms emit a strong orange fluorescence that can be detected separately from the red fluorescence emission of their chlorophyll [43], [76]. Prochlo cells were distinguished by the presence of red florescence and the lack of orange fluorescence. Bac were identified as being DNA positive events lacking both red and orange fluorescence.

Data for natural samples are typically collected for 2 to 4 minutes with a flow rate of 50 to 100 µl min^−1^ [77]. All samples were run at a flow rate of 100 µl min^−1^; this flow rate was provided from the BD Service Engineer considering the machine specifications, and we also did a manual check and measured the amount of waste flowing out in one minute into a 50 ml tube which determined the flow rate for the setting and pressure we were working on. The analysis time was recorded to precisely determine the cell concentrations of each type of picoplankton. The absolute cell concentrations for each population in a given sample were calculated as follows:

*C*_pop_ = (*V*_total_/*V*_sample_) * *N*_pop_/(T*R) Where: *C*_pop_ is the concentration of picoplankton in cells µl^−1^; *V*_total_ is the volume of sample in µl plus additives (fixatives, dyes, beads, etc.); *V*_sample_ is the volume of sample analysed in µl; *N*_pop_ is the number of cells acquired; *T* is the acquisition time in min; *R* is the sample flow rate in µl min^−1^ [77].

### Measurement of sponge pumping rates

Pumping rate estimations were performed during the sampling days through dye-release experiments. Sponges were filmed *in situ* and sodium fluorescein dye was released next to the sponge. The pumping activity of three specimens of each species was visualised and recorded by releasing dye at the base of the specimen and observing the movement of the dye through the sponge. A ruler was placed next to the sponge specimen and used as a scale reference in the field of view of the camera. Subsequent frame-by-frame image and video analyses were performed to estimate pumping velocity, where only frames showing the vertical movement of the dye through 2 cm of water immediately above the osculum were used to measure the distance travelled by the dye-plume per unit time [59]. Two oscula per specimen of each of the seven study species were used for the pumping rate calculations (6 measurements for each species in total). Our flow rates for an individual osculum were comparable to those in the literature [78].

Volume flux or pumping rate (Q), which is the volume of water exiting an osculum per unit time, was calculated by multiplying the exhalant flow speed (*v*) expressed in cm s^−1^, by the cross-sectional area of the osculum (A), using the equation: Q = *v*A. This calculation assumes plug flow which is most likely true for sponges [10], [59]. Volume flow rate (the total volume of water processed per unit time, s^−1^) was then estimated by multiplying the pumping rate (Q) by the number of oscula per sponge, as the study species are all multi-oscular sponges [59]. This provided an estimation of the total volume of water processed by each sponge.

### Retention efficiency and number of cells filtered

Retention efficiency, expressed as the percentage of picoplanktonic cells removed by three specimens of each of the study species from inhalant water samples, was calculated as: 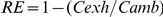 Where *Cexh* is the concentration of cells in the exhalant water and *Camb* is the concentration of cells in the ambient water. Then, the number of cells filtered was calculated by multiplying: retention efficiency (no units), volume flow rate (ml s^−1^) and ambient concentration of cells (cells ml^−1^), as described by Trussell *et al.* [10]. All means are presented with standard deviations.

### Carbon flux estimations

Estimates of particulate organic carbon (POC) from the picoplanktonic organisms were estimated using the mean number of cells removed per ml^−1^ by each sponge, as determined by flow cytometric analysis. This value was then converted to mg of C for each type of picoplankton using the following standard cell conversions from the literature: non-photosynthetic bacteria, 20 fg C cell^−1^ [79]; *Prochlorococcus* sp., 61 fg C cell^−1^ [80]; *Synechococcus* spp., 178 fg C cell^−1^ [81]. These conversions were used because they were calculated for cells with mean diameters that correspond to the cell diameters found during our study [30], which were visually confirmed using confocal microscopy. For each sponge specimen, carbon acquired per second was calculated by multiplying the number of cells retained (cells ml^−1^ s^−1^) by the quantity of carbon contained in each type of cell [10]. The data are presented in such a way that POC fluxes can be re-calculated if more accurate carbon equivalents become available for the picoplanktonic organisms specific to the study area.

### Sponge abundance and study area calculations

The calculations above provide estimates of the carbon consumed by individual sponges per unit time; however the intention was also to estimate the amount of POC consumed by a sponge assemblage. For this purpose, the abundance of sponges, the volume of water in a known area (Taputeranga marine reserve), and the amount of POC contained within the water (based on the data from the ambient water), were estimated.

The Wellington south coast supports diverse sponge assemblages with up to 500 sponges per m^2^ in some areas, covering over 50% of the substratum at some sites [82]. At the study site, sponge percentage cover and sponge density have been previously estimated from 0.5 m^2^ photoquadrats [82] for the most abundant species, including the species selected for the present study. The results from these earlier surveys showed that sponge coverage is highly variable; therefore, a range of values for sponge percentage cover was used for all seven species combined. In order to account for the high variability in sponge abundance in the study region and because it was not possible to sample the entire reserve area, low, mid and high estimates (0.1, 0.5, 1, 1.5 and 5%) of sponge coverage were used based on the coverage calculated for the study species living on vertical rock walls. The different values of sponge coverage were used for subsequent calculations and all the characteristics (diet composition, sponge abundance, feeding efficiency, pumping rate and number of food particles removed) analysed from the seven study species were used as a representation of the sponge assemblage for the given range of sponge abundances. To integrate the information of the amount of POC consumed by individual sponges to estimate the amount of POC consumed by a sponge assemblage, calculations were made using the measured sponge areas of the study species and by assuming a uniform sponge thickness of 1 cm (which is based on field observations of the species).

To estimate the volume of water in the reserve, we compiled information on bathymetry and total rocky reef area from habitat maps of the study region [83]. We binned the area into several regions and then calculated the total volume of water in m^3^ based on the average depth for the region using the different depth ranges from the bathymetry maps. Similar calculations were performed using the submarine rocky area to estimate the total area of reef in the reserve expressed in km^2^. To estimate the percentage of picoplanktonic POC removed by the sponges from the total available in the water column, we assumed a homogeneous distribution of bacterioplankton throughout the water column. In a coastal turbulent environment such as the one studied here, there is likely to be enough mixing by wave action to make the first 10 m or so homogenous. This has recently been confirmed by ongoing studies at Victoria University of Wellington in the same area, where chlorophyll records showed no variation between 0–10 m depths (César A. Cárdenas personal communication).

### Supporting calculations

Based on the calculated dimensions of the study area, and using the data from all seawater samples collected, the ambient number of picoplanktonic cells present in the volume of water in the study area at any one time was calculated as:
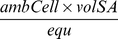
(1)Where: *ambCell* is the ambient concentration of cells (cells ml^−1^), *volSA* is the volume of water in the study area (m^3^) and *equ* is the equivalent of 1 ml in m^3^ (0.000001). Accordingly, the number of cells that sponge assemblages would be capable of removing on a daily basis in the study area (assuming that sponges were actively pumping for 24 hours per day) was estimated using a variation of the previous equation:
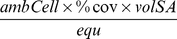
(2)Where *%cov* are the different values of sponge percentage cover (based on estimates of abundance).

The amount of POC acquired per day (obtained from the carbon conversions) by the individual study species was included to calculate the average amount of POC consumed for the different values of sponge coverage in the study area per day according to the following equation:
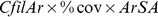
(3)Where *CfilAr* is the POC filtered (mg C d^−1^) per unit area of sponge, *%cov* is the sponge percentage cover (0.5 to 5%), and *ArSA* is the total rocky reef area in the reserve (m^2^). To obtain the *CfilAr*, the carbon acquired d^−1^ per sponge (with three specimens for each species) and the area (cm^2^) of each sponge specimen, were divided to obtain the POC consumed normalised per unit area of sponge. Finally, since the ambient cell concentration (cells ml^−1^) for the three picoplanktonic organisms detected is known, along with the amount of carbon present in each type of cell (from the carbon conversions), it was possible to calculate the total amount of POC available in the study area as a result of the three groups of picoplankton. Using this value, the proportion of the total POC pool being consumed by the sponge assemblage in the reserve was estimated with the following equation:
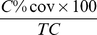
(4)Where *C%cov* is the amount of prokaryotic POC consumed in the study area for the different values of sponge percentage cover (0.5 to 5%) and *TC* is the total amount of prokaryotic POC (g C d^−1^) available in the study area estimated from the ambient cell concentrations for all the study species.

### Data analysis

#### Cell concentrations

For each sponge species, a Generalised Linear Model (GLM) was used to conduct an analysis of deviance with a quasibinomial error distribution (to correct for over- dispersion) and a log-link function to model inhalant cell concentration against exhalant cell concentration, and type of picoplankton (three levels: ‘Bac’, ‘Prochlo’, ‘Synecho’). Likelihood ratio tests were used to examine the hypothesis that a significant interaction occurred between the inhalant and exhalant water and picoplankton (in all cases *P*<0.05). In the absence of significant interactions, the interaction term was removed and we concentrated on the main effects of inhalant-exhalant and picoplankton.

### Retention efficiency

A one-way analysis of variance (ANOVA) was used to model retention efficiency (percentage retained between inhalant and exhalant water) against the type of picoplankton (three levels: Bac, Prochlo, Synecho). The percentage data (retention) were arcsine and square root transformed to meet assumptions of normality and equal variance. The assumption of homogeneity of variance was examined using Bartlett's test (*P*>0.05 in all cases). For the significant main effects (P<0.05), Tukey's HSD was used to examine pairwise comparisons and data were pooled (from the three specimens) for the final calculations. Statistical differences were determined at the 5% level and all statistical analyses were conducted by R ver. 2.10 [84].
